# VisuaLife: library for interactive visualization in rich web applications

**DOI:** 10.1093/bioinformatics/btab251

**Published:** 2021-05-07

**Authors:** Justyna D Kryś, Dominik Gront

**Affiliations:** Faculty of Chemistry, Biological and Chemical Research Center, University of Warsaw, Warsaw 02-093, Poland; Faculty of Chemistry, Biological and Chemical Research Center, University of Warsaw, Warsaw 02-093, Poland

## Abstract

**Motivation:**

Visualization is a powerful tool to analyze, understand and present big data. Computational biology, bioinformatics and molecular modeling require dedicated tools, tailored to very complex, highly multidimensional data. Over the recent years, numerous tools have been developed for online presentation, but new challenges like the COVID-19 pandemic require new libraries which will guarantee fast development of online tools for a better understanding of biomedical data/results.

**Results:**

VisuaLife is a Python library that provides a new approach to visualization in a web browser. It offers 2D and 3D plotting capabilities as well as widgets designed to display the most common biological data types: nucleotide or protein sequences, 3D biomolecular structures and multiple sequence alignments. Components provided by the VisuaLife library can be assembled into a web application to create an analysis tool tailored to provide multidimensional analysis of a specific research problem. VisuaLife, to our best knowledge, is the most modern solution that allows one to implement such a client-side interactivity in Python.

**Availability and implementation:**

The git repository of the library is hosted at BitBucket: https://bitbucket.org/dgront/visualife/. PyPI distribution is also provided for MacOS and Linux. While basic examples are provided in the supporting materials, the full documentation is available at ReadTheDocs website: https://visualife.readthedocs.io/.

**Supplementary information:**

Supplementary data are available at *Bioinformatics* online.

## 1 Introduction

Visualization plays a very important role in science. This is especially true in the case of biological observations, which typically are very complex and highly multidimensional. In recent years, more and more bioinformatics tools have been devised to present data online, directly from a cloud to a web browser. Such solutions, termed *rich* or *progressive web applications* require neither installation of any additional programs nor downloading data to a client machine. A number of visualization toolkits have been published recently, such as Bio.js: Yachdav *et al.* (2015), GenomeSpace: Qu *et al.* (2016) and many others Wang *et al.* (2015). All these toolkits are written in JavaScript, as currently, this is the only language that ensures interactivity in a web browser. JavaScript, however, is rarely used in a broad academic research. The use of such dedicated frameworks can be very helpful however their adaptation, customization and merging into a single web application is still a highly non-trivial task that requires an expert knowledge that is generally not available to a typical researcher.

On the other hand, Python has emerged as an extremely popular language in data science, machine learning and general-purpose scripting. Therefore, we decided to develop a software library, implemented in Python, that will allow visualization of biological data and lower the barrier of making interactive presentations without the need to write any JavaScript code (although JavaScript can be very easily combined with VisuaLife).

## 2 Materials and methods

### 2.1 Implementation

VisuaLife is an object-oriented Python library released under the Apache2 license. The library has been designed to work both on the server-side (e.g. on a desktop computer, in a terminal, etc.) and on the client-side (i.e. in a web browser, as a part of an HTML page). Scripts written for one of the two environments are nearly identical to those working in the other. To achieve that, we implemented a hierarchy of viewports, i.e. objects responsible for rendering graphics. Scripts that run in a terminal use SVGViewport and produce vector graphics in SVG file format; scripts that are executed on a client (i.e. in a web browser) use HtmlViewport. Raster graphic is displayed in a web browser window with CanvasViewport. All the viewports share the same interface and provide the same methods to draw basic shapes as like lines, circles, rectangles, etc. VisuaLife scripts executed by a web browser use Brython (https://brython.info) to seamlessly translate Python to JavaScript. Brython provides methods to access elements on a webpage so a user can, e.g. change styles or bind events to every element that has a known unique identifier. Therefore, the vector graphics created by VisuaLife can be easily made interactive by adding the appropriate Python code. VisuaLife can read several file formats commonly used in bioinformatics and molecular modeling like MOL2, HSSP, PDB, FASTA, MSF or ALN. This I/O functionality can be used both by local scripts to load input files from a disk and in a web browser when an input file is dropped on a web application window.

### 2.2 Widgets

Widgets are interactive web page elements that are used to display given data in a browser, such as biomolecular sequences, structures and their alignments. From a programmer’s perspective, a widget is an object of a given class; the API of that class (i.e. its methods) provides the necessary functionality to operate on given data. More than one object of the same class (e.g. protein sequences displayed as SequenceViewer instances) can be displayed by a single page; objects are then identified by the ID of the enclosing DIV element of that page. A context drop-down menu, which may be attached to a widget, allows a user to manually rather than programmatically control the widget’s behavior. Besides displaying biological data, VisuaLife also provides a few general-use widgets. All of them have been listed in Table 1.

**Fig. 1:**
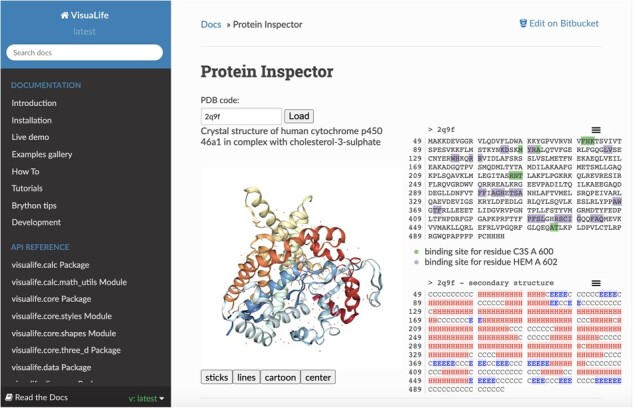
Protein Inspector application with VisuaLife widgets: SequenceViewer, SecondaryStructureViewer, StructureViewer, TooltipWidget and AjaxSender. Interactivity that binds these widgets is included in the HTML page as Python functions

**Table 1:** Widgets available from the VisuaLife library

General-purpose widgets	
FileReaderWidget	Reads a file on drag and drop action
TableWidget	Creates a table
TooltipWidget	Shows tooltip on a mouseover event
AjaxCommunication	Used for client-server communication
Data-specific widgets	
SequenceViewer	Displays nucleic acid or protein sequence
SecondaryStructure Viewer	Displays protein secondary structure
SequenceFeaturesBar	Displays annotations along a sequence
MSAViewer	Displays multiple sequence alignment
StructureViewer	Displays 3D structure using NGL library

### 2.3 Distribution and documentation

The repository with the source code is publicly available on BitBucket. PyPI distribution is also provided for MacOS and Linux. Documentation, which can be found on the visualife.readthedocs.io website, provides a detailed description on installation and use of the software, API documentation, tutorials and a gallery of examples. Every example in that section is a stand-alone HTML page that displays graphical content; the majority of them are interactive. The examples have been also published on the CodePen website where one can experiment with them without any installation required. Finally, we provide a web page with an interactive console with the VisuaLife library included that serves as a library testing ground. Along with VisuaLife, we also built a simple *Protein Inspector* example application that has been described in details in the Tutorial section of VisuaLife documentation. The application displays a 3D structure, sequence and secondary structure of a chosen protein. Interactivity between these three widgets allows a user to analyze all these aspects at one time. All data are downloaded from the Internet (https://www.rcsb.org and https://www.ebi.ac.uk/) with REST services.

## 3 Conclusion

In this contribution, we present a library of widgets: web page components that are devised to interactively present biological data online. Plots and widgets can be easily combined into a web application tailored to the user’s data. Interactivity, e.g. respective callback methods as well as program logic that glues these components into a web application can be easily achieved by adding simple Python code to a web page. We believe our choice of programming language will make a big difference as online visualization will no longer be restricted to JavaScript experts. The server-side part of a web application can also be implemented in Python, e.g. with the help of bioinformatics frameworks like Biopython: Chapman and Chang (2000) or Bioshell Macnar *et al.* (2020). Given the widespread popularity of the Python language among life scientists we hope VisuaLife will become a very popular tool.

*Financial Support*: This work was supported by the Polish National Science Centre (NCN) 2018/29/B/ST6/01989.

*Conflict of Interest*: none declared.

## Supplementary Material

btab251_Supplementary_Data

## References

[btab251-B1] Chapman B. , Chang J. (2000) Biopython: python tools for computational biology. SIGBIO Newsl., 20, 15–19.

[btab251-B2] Macnar J.M. et al (2020) BioShell 3.0: library for processing structural biology data. Biomolecules, 10, 461. 32188163 10.3390/biom10030461 PMC7175226

[btab251-B3] Qu K. et al (2016) Integrative genomic analysis by interoperation of bioinformatics tools in GenomeSpace. Nat. Methods, 13, 245–247. 26780094 10.1038/nmeth.3732 PMC4767623

[btab251-B4] Wang R. et al (2015) Open source libraries and frameworks for biological data visualisation: a guide for developers. Proteomics, 15, 1356-74 10.1002/pmic.201400377 PMC4409855 25475079

[btab251-B5] Yachdav G. et al (2015) Anatomy of BioJS, an open source community for the life sciences. eLife, 4, 1–7. 10.7554/eLife.07009 PMC4495654 26153621

