# Supplementary material for: VisuaLife: library for interactive visualization in rich web applications
Source: Bioinformatics. 2021 May 7;37(20):3662–3. doi: 10.1093/bioinformatics/btab251 (PMC8136008; doi:10.1093/bioinformatics/btab251)
Supplement: btab251_Supplementary_Data [file btab251_supplementary_data.pdf]

Supplementary material

The `widget` module of VisuaLife library provides components devised to visualise biomacromolecular structure and sequence. This document gives a brief overview of the functionality that is currently available. For the most recent documentation refer to the `https://visualife.readthedocs.io` website. Code snippets provided here where taken from two example applications: *„Protein inspector”* and *„Ab - spikes”*, which are available from VisuaLife documentation[1],[2]. The *„Protein inspector”* page shows basic information about a PDB deposit; *„Ab - spikes”* visualises interactions between a Covid-19 spike protein and antibody chains.

1 VisuaLife widgets

Currently the library provides widgets that can be used the following bioinformatic data:

- protein and nucleic sequences
- protein secondary structure
- multiple sequence alignments
- sequence annotations (e.g. binding sites)
- biomolecular 3D structures

Additionally the library provides generic widgets that facilitate creating a web application, such as the `AjaxCommunication`, to ease client-server communication, loading data from disk, etc.

2 Python and a web browser

VisuaLife uses Brython[3] to run Python code in a web browser environment. Brython provides a dictionary-like access to any web page element, e.g. when a button and a text input element are defined as follow:

[1] `https://visualife.readthedocs.io/en/latest/doc/protein_inspector.html`
[2] `https://visualife.readthedocs.io/en/latest/doc/ab_spikes.html`
[3] `https://brython.info`

```
<input type="text" id="pdb_name" name="pdb_name" value="2q9f">
<button id="load" class="button">Load</button>
```

in a Python code one can interact with these elements accessing them by their id string that has been assigned in the HTML part:

```
document["load"].bind("click", download_data)
pdb_name = document["pdb_name"].value
```

`document` is the name of the dictionary holding the page content that has been automatically created by Brython.

3 Loading and parsing data

`AjaxCommunication` widget may be used to load user's data into a web application. It requires a URL as the first argument and a name of the call-back function that is executed after the data has been received. Note, that unlike all other existing Web frameworks, the call-back procedure is a regular Python function. When the data to be loaded is located on a local computer, the URL should be replaced with a path to this file:

```
get_msa = AjaxCommunication("az.fasta", receive_msa, "GET")
```

where `"az.fasta"` is the name of the local file and `receive_msa` is the name of the procedure that will be executed upon receiving the data. Finally, "GET" is an optional argument that indicates request type ("POST" is the default). The details about HTTP request types can be found on W3School website[4] The following example uses only data and services that are publicly available from: `https://www.ebi.ac.uk` and `https://www.rcsb.org`:

```
pdb_name = document["name"].value
pdb_ajax = AjaxCommunication("https://files.rcsb.org/view/%s.pdb" %
    pdb_name, structure)
pdb_ajax()
AjaxSender("https://www.ebi.ac.uk/pdbe/api/pdb/entry/summary/%s" \
    % pdb_name, receive_protein_description, "GET")()
```

Upon receiving a response, a respective callback function is called. That function needs to decode the data received as a `HttpRequest` object which is the only argument of the function. Typically it uses `json.loads()` Python utility that converts JSON string into Python data structures such as lists and dictionaries. In the following example the basic information about a PDB deposit is received from EMBL RESTfull service:

[4] `https://www.w3schools.com/tags/ref_httpmethods.asp`

```python
def receive_protein_description(req):

    data = json.loads(req.text)
    description = data[pdb_name][0]["title"]
    document["loading"].text = description.lower().capitalize()
```

4 Creating a widget

Once data has been downloaded and processed, one can easily display it with an appropriate widget, such as a SequenceViewer.

```python
def display_sequence(evt):
    global seqview, seq

    seq = read_fasta(evt.text)[0]["sequence"]
    seqview = SequenceViewer("show_sequence", "7cwl A", seq, \
          n_columns_of_ten=4)
    seqview.click_on_sequence_callback = click_on_aa
    seqview.menu.add_menu_option("B-factor average",avg_callback)
    seqview.menu.add_menu_option("H-bonds",hbonds_callback)
    seqview.menu.add_menu_option("stacking",stacking_callback)
    seqview.menu.update_menu()


    AjaxCommunication("7cwlA.fasta", display_sequence, "GET")()
```

5 Interactions between widgets

The above code snippet illustrates also how to add interactions to page elements. VisuaLife widgets provide methods to build a menu for a component. A menu entry can be linked to a user-defined Python function, defined in the page's script section. Another possibility to connect a page element with a function is to use `bind` method provided by Brython.

```python
document["load"].bind("click", download_data)
```

In the example above a Python function named `download_data` is bound to an HTML button; it fill be fired on "click" event.
